# Worldviews and Values as Bases for Political Orientations

**DOI:** 10.5334/irsp.741

**Published:** 2023-04-10

**Authors:** Girts Dimdins, Henry Montgomery, Maria Sandgren

**Affiliations:** 1 University of Latvia, Latvia; 2 Uppsala University, Sweden; 3 Mälardalen University, Sweden

**Keywords:** political orientation, basic personal values, political values, worldviews, ideology, structural equation modelling

## Abstract

This study used structural equation modelling to test how political orientations (self-reported placement on socially liberal-conservative and economically left-right continuums) could be predicted from basic worldviews (a generally humanistic vs. normativistic orientation) and basic personal values (concepts pertaining to desirable end states or behaviors) in conjunction with core political values (normative principles about functioning of society) in a population sample from Sweden. In general, political orientation was much more strongly predicted by social-focus values (conservation and self-transcendence), and only weakly predicted by personal-focus values (self-enhancement and openness to change). The results also showed that basic personal values in conjunction with core political values mediated the relationship between basic worldviews and political orientation.

Since the turn of the century, psychology and political science have developed a steadily growing interest in the study of the psychological underpinnings and correlates of political orientation and its behavioral consequences (Azevedo et al., 2019; Beattie et al., 2021; Cichocka & Dhont, 2018; Feldman & Johnston, 2014; Jost et al., 2009; Zmigrod, 2022). Researchers have increasingly focused on identifying and explaining the systematic differences in the psychological functioning of people who identify, or can be identified, as liberal versus conservative, or rightist versus leftist. Two interconnected lines of research and theoretical argumentation have emerged as central to the study of political orientation as a psychological construct. One line focuses on the very structure and conceptualization of political orientation, such as whether it should be conceived as a unidimensional construct with two opposing poles (such as liberal vs. conservative), or a multidimensional construct with different facets that can be interrelated in various ways, such as socio-cultural, economic, and religious aspects of political orientation (Choma et al., 2012; Feldman & Johnston, 2014; Von Collani & Grumm, 2009). The other line of research looks at how various psychological constructs (such as individual traits, motivational factors, cognitive styles, etc.) fit in the structure of political orientation and how particular psychological constructs determine, correlate with, and depend on the individual’s political orientation (Braithwaite, 1998; Caprara et al., 2009; Dimdins et al., 2016; Jost, 2009; Nilsson et al., 2020; Schwartz et al., 2010).

This paper attempts to contribute to both lines of research, with its primary focus on examining the relation of values and worldviews to the political orientation. In developing this model, we have relied on two theoretical frameworks that should be related to political orientation, both on the basis of theoretical reasoning and the previous empirical studies. One framework is Schwartz’s (1992) theory of basic personal values (Schwartz, 1994; Schwartz et al., 2010). The other one is the polarity theory, originally formulated by Tomkins (1965) and more recently developed and refined by Nilsson (2014), which has been proposed as a possible psychological basis of political orientation (Nilsson & Jost, 2020). We have integrated the theoretical insights from these two theories with findings from the previous empirical work pertaining to the structure of political orientation to refine and test a comprehensive psychological model of political orientation.

## Structure of Political Orientation

We define political orientation as a self-reported summary expression of an individual’s system of social, political, and economic beliefs about proper functioning and organization of society. In political and social psychology literature, the term *political ideology* is often used, with the same meaning to describe the same psychological construct (see, e.g., Jost, 2006; Jost et al., 2009), and the two terms are often used interchangeably as synonymous (Zmigrod, 2022). In our writing we use the term *political orientation* to refer to the individual-level measurement of the summary of individual’s ideological beliefs, but we use the term ‘ideology’ to refer to the political belief systems as such. The theoretical conceptualization (and, correspondingly, operationalization, or psychological measurement) of political orientation is a complex problem that has been much discussed in the literature (Feldman & Johnston, 2014; Zmigrod, 2022). Alternative conceptualizations of ideology have been suggested in the literature, ranging from a simple left-right (or liberal-conservative) continuum to multidimensional models built on various social and political values and attitudes. A simple one-dimensional continuum, often based on individual self-labeling in terms of liberalism-conservatism (or leftism/rightism), is a commonly used method for measuring political orientation and appears to work relatively well in societies with well-developed political cultures and political discourses (e.g., Western Europe and Anglo-Saxon countries), where these terms have well-defined and commonly shared meanings (Jost, 2006); it should be noted, though, that also among these ‘old’ democracies significant between-country differences can be observed in terms of people’s ability to self-identify on the left-right continuum, especially among youth (c.f. Mieriņa, 2018). Some studies using one-dimensional measurement of political orientation have found complex structures of values, attitudes, and political party preferences underlying this orientation (Caprara et al., 2009; Nilsson et al., 2020; Schwartz et al., 2010; Von Collani & Grumm, 2009).

Perhaps the most commonly used model in studies of political orientation today is based on a two-dimensional conceptualization of ideology (Braithwaite, 1998; Feldman & Johnston, 2014; Jost, 2006). The first dimension corresponds to acceptance versus rejection of social change, and the second dimension corresponds to rejection versus acceptance of social inequality, especially economic inequality (Jost et al., 2009). The first dimension has also been conceived as the social (or cultural) dimension of political ideology, with endorsement of traditional values and maintaining the existing societal structure representing one end of the continuum, and facilitation of social change and augmenting the rights and freedoms of all individuals representing the other end. The latter end of the continuum can be referred to as ‘socially liberal’, whereas the former can be labeled ‘socially conservative’ (Feldman & Johnston, 2014). The second dimension can be conceptualized as the economic dimension of ideology, where one end of the dimension stands for increasing economic equality in society by redistributing incomes and resources, and the other end represents a meritocratic position where everyone is responsible for the outcomes of their economic activity and their economic well-being. This dimension has been labeled ‘economic liberalism vs. conservatism’ (Feldman & Johnston, 2014), or ‘economic leftism vs. rightism’ (Caprara et al., 2009). A comparison between a one-dimensional and two-dimensional operationalization of political orientation has demonstrated a greater explanatory power (in terms of predicting political attitudes) of the two-dimensional approach (Von Collani & Grumm, 2009). Both dimensions have been shown to robustly correlate in Western democracies in such a way that higher social conservatism tends to coincide with higher economic rightism, and social liberalism with economic leftism (Prosser, 2016). However, cross-cultural comparisons have revealed that the relationship between the two dimensions is not consistent in different societies, showing positive, negative, and non-existent correlations in Eastern European countries and other non-Western cultures characterized by lower socio-economic development and lower ideological constraints (Choma et al., 2012; Cochrane, 2010, 2011; Malka et al., 2014). The concept of ideological constraint relates to the extent to which various political beliefs and attitudes tend to be structured along the ideological spectrum (left vs. right or liberal vs. conservative) and reflects the contents of political discourse within a society or its subgroup(s) (Converse, 1964). In societies with high ideological constraints (e.g., the Netherlands, Sweden, United States) the public discourse prescribes which beliefs and attitudes correspond to each of the end-poles of the ideological spectrum and thus should be positively or negatively related, whereas in societies with low ideological constraints (e.g., Chile, Indonesia, Slovenia) the relationships among political beliefs and attitudes are less systematic and more variable (all examples from Malka et al., 2014, supplementary material). These findings suggest that both dimensions are structurally and functionally independent, and differentially related to various psychological variables such as need for security, need for cognition, or egalitarianism (Cochrane, 2010, 2011; Feldman & Johnston, 2014).

## Basic Personal Values

One theoretical framework that has been used to explain the psychological structure of political orientation is the theory of basic personal values (Caprara et al., 2009; Piurko et al., 2011; Schwartz et al., 2010). Personal values are concepts or beliefs pertaining to desirable end states or behaviors that cut across specific situations, guide the selection or evaluation of behavior and events, and are ordered by their importance (Schwartz, 1992). In the most widely used formulation of his theory, Schwartz (1994) and Schwartz et al. (2010) distinguish between ten value types (or basic values): power, achievement, hedonism, stimulation, self-direction, universalism, benevolence, tradition, conformity, and security. When the correlations among these ten basic values are visualized, for example, with the help of a multidimensional scaling analysis, the basic values tend to form a circular structure, with the same order of values around the circle, and this order has been shown to be universal across many cultures. In this circular structure, the adjacent values tend to be positively correlated, and the values on the opposite sides of the circle show weaker correlations than those of adjacent values, or these values can be uncorrelated or negatively correlated. The ten basic values can be further reduced to two fundamental value dimensions, orthogonally cutting through the value circle. The first dimension is self-enhancement versus self-transcendence, with such values as universalism and benevolence corresponding to the self-transcendence end of the dimension, and power and achievement forming the self-enhancement end (Schwartz, 1994). The other dimension is conservation (which includes the basic values of tradition, conformity, and security) versus openness to change (with the corresponding values of self-direction and stimulation) (Schwartz, 1994). The end-poles of these dimensions can be seen as higher-order psychological constructs, around which the ten values are robustly organized, and we used these end-poles to structure our theoretical model described below. In addition, conservation and self-transcendence values can be characterized as social values (with their focus on serving the interests of specific groups and society in general), whereas self-enhancement and openness to change values can be considered personal values (focusing on serving the interests of the individual). Self-transcendence and openness to change values together represent growth-oriented values, and conservation and self-enhancement values represent self-protection values (Schwartz et al., 2012).

## Basic Worldviews

Polarity Theory, originally proposed by Tomkins (1965) and more recently refined by Nilsson (2014), postulates that there are generalized worldviews that are associated either with freedom and humanism, or with regulation and normative concerns. The former worldview is defined as humanism, which presents humans as essentially good and their experiences as innately valuable, and which sees the purpose of society as facilitating human growth. The latter worldview is defined as normativism, and it portrays human beings as essentially bad and in need of external rules and norms to be prevented from engaging in irresponsible and harmful behavior (Nilsson, 2014). Each of the basic worldviews (humanism and normativism) can further be conceptualized into five tenets: human nature (humans seen as good vs. bad), interpersonal attitude (emphasizing warmth vs. discipline), attitude to affect (representing trust and openness vs. obedience and control), political values (focusing on rights and well-being vs. law and order), and epistemology (seeing roots of knowledge in creativity and enjoyment vs. rigorous empiricism and minimization of error). Polarity theory has been suggested as a possible theoretical mechanism explaining the psychological foundations of political orientation; according to this theorizing, the basic worldviews as generalized orientations acquired early in life predispose individuals to accept the ideas of liberal/left-wing versus conservative/right-wing ideologies (Jost et al., 2003; Tomkins, 1995). Until recently, this theoretical account had limited empirical support, but there has been empirical evidence emerging in support of it (Dimdins et al., 2016, Nilsson & Jost, 2020), indicating the need for further research into the psychological mechanisms relating basic worldviews to political orientation.

Measurements of normativism and humanism correlate with multiple psychological constructs in a pattern consistent with the notion of broad, generalized, basic worldviews. For example, normativism has been shown to be positively correlated with lay essentialism, entity theory about the world, determinism, cynicism (Nilsson & Strupp-Levitsky, 2016), and binding moral foundations (Dimdins et al., 2016; Nilsson & Strupp-Levitsky, 2016), whereas humanism is positively correlated with subjectivist epistemology, organicism, supernatural beliefs, anti-physicalism, societal constructionism, religious universalism (Nilsson & Strupp-Levitsky, 2016), individualizing moral foundations, and interpersonal trust (Dimdins et al., 2016; Nilsson & Strupp-Levitsky, 2016). Normativism has been shown to predict voting preference for a nationalist party, but not for conservative/right-wing parties in general (Nilsson et al., 2020). Most importantly for our model, both worldviews have been shown to correlate with ideological beliefs: in Nilsson and Jost’s (2020) findings, normativism was robustly associated with rightist/conservative self-placement, resistance to change, acceptance of inequality, and a number of psychological variables related to uncertainty aversion and threat sensitivity. Humanism was strongly associated with preferences for equality and openness to change. These findings allow us to put the two worldviews at the basis of our theoretical model. They represent individuals’ general inclination to see people as generally good (humanism) or generally bad (normativism), which can be further manifested in a variety of ways and contexts. Thus we hypothesize that these worldviews form a broad psychological basis for the value orientation factors in our model (to be described below), which can be expressed in other, more specific psychological constructs, such as values, core political values, and political orientation. Previous research has revealed positive links between humanism and self-transcendence values, positive relationships between normativism and self-enhancement values as well as conservation values, and negative relationships between normativism and self-transcendence values (Carlson & Levy, 1970; de St. Aubin, 1996; Dimdins et al., 2016; Nilsson & Strupp-Levitsky, 2016).

In examining the relation of basic worldviews to political orientation, our study complements a recently published study by Nilsson and Jost (2020) that examined how a number of seemingly disparate psychological constructs, such as epistemological orientations, and beliefs about human nature and the social world, are related to political left-right orientation. As is true in Nilsson and Jost’s (2020) study, our goal is not to establish a causal model of how humanism and normativism relate to other variables. Rather we used structural equation modeling as a tool for exploring the extent to which humanism and normativism may function as unifying constructs for predicting the associations between the value orientation factors and how they are further related to basic personal values, core political values, and political orientation.

## Core Political Values

Core political values (CPVs) can be defined as general normative principles and suppositions about how government, citizenship, and society should function. These values serve as general focal points for taking positions on various social, economic, and political matters in potentially confusing political environments (Schwartz, 2006). CPVs have been shown to mediate the relationship between basic personal values and voting behavior (Schwartz et al., 2010). There is no universal theory regarding the content (or number) of CPVs in contemporary democratic societies. Schwartz et al. (2010) have suggested one of the most comprehensive models of core political values, which includes such values as traditional morality, blind patriotism, law and order, foreign military intervention, free enterprise, equality, civil liberties, and accepting immigrants. In comparison with the basic worldviews (which pertain to basic views of human nature) and basic personal values (which pertain to basic convictions of what is good and desirable), CPVs are more specifically geared towards evaluation of the political environment and the political functioning of a society. They can be seen as basic ideological convictions, forming the bases of corresponding networks of political attitudes in various life domains of contemporary societies.

## The Hypothesized Model

**Figure 1:**
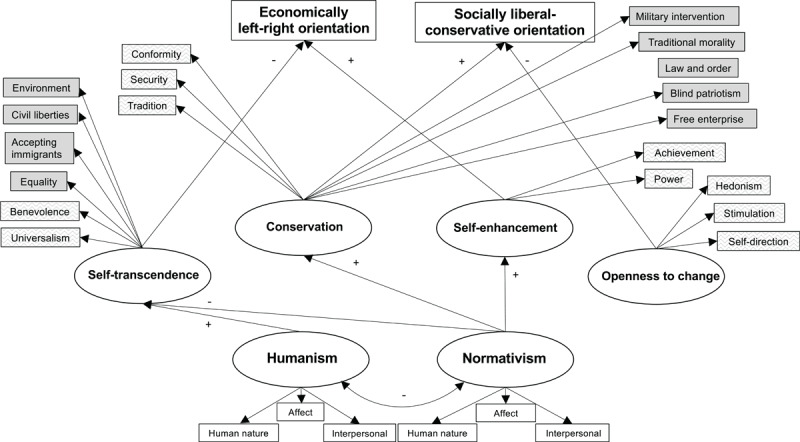
The base model for connections between worldviews, basic personal values (lighter gray boxes) and core political values (darker gray boxes). Political values are linked to value orientations in line with data from Schwartz et al. (2014).

In this study we combine insights from the three lines of literature outlined above to examine the structure of political orientation. Our model is built around the two dimensions forming the structure of basic individual values: conservation versus openness to change, and self-enhancement vs. self-transcendence. However, in our model we extend the psychological function of these value dimensions to go beyond basic personal values. We use the end-poles of these dimensions as conceptual value-orientation factors, related to basic personal values, core political values, basic worldviews, and political orientation. We test the mediating role that these value orientation factors (manifested in both basic values and core political values) play in the relationship between worldviews and political orientation. In addition, we contribute to the studies of the relationship between values and political orientation (Schwartz et al., 2014) by discovering and directly testing links not revealed in multidimensional scaling (e.g., negative relationships among the studied variables). In our model we test the possibilities for predicting political orientations from the value orientation factors and worldviews. In particular, we hypothesize that political orientations can be predicted from worldviews through value orientation factors as mediators. The model proposes links from basic worldviews to the four value orientation factors, and further links from the value orientation factors to basic values, core political values, and the two direct measurements of both dimensions of political orientation (social liberal vs. conservative and economically left vs. right orientation). The model implies that we expect to find four latent variables (corresponding to the four value orientation factors – conservation, openness to change, self-enhancement, and self-transcendence), with the associated basic values, core political values, and measurements of political orientation as indicators, that are differentially predicted from humanism and normativism. Note that we do not use basic values, core political values, and political orientations predicted from the same value orientation factor as items for measuring that value orientation factor as a single construct; rather, we examine how the latent variables corresponding to the four value orientation factors can be used to simultaneously predict basic values, CPVs, and political orientations. To assign basic personal values as indicators to value orientation factors, we relied on Schwartz’s model, from which the value orientation factors were originally derived. To assign core political values as indicators to value orientation factors, we followed the results on the relations between basic personal values and core political values reported by Schwartz et al. (2014) in a multidimensional scaling analysis of data from 12 European traditional democracies (p. 919). Figure 1 provides a summary of the theoretical model that served as the basis for designing this study.

The model in Figure 1 includes only positive links between core political values and value orientation factors, which were clearly visible in Schwartz et al.’s (2014) multidimensional scaling results. Negative correlations between core political values and personal values that were predicted by Schwartz et al. (2014) were mostly confirmed in their study, but were, on the whole, very close to zero, and we saw it as an open question whether negative links would turn out to improve the fit of the model. We also saw it as an open question whether additional positive links could be added to the model. For this reason, we used structural equation modelling to first test the model in Figure 1 (which we will denote as the base model) and then investigated in a systematic way the extent to which the model could be refined (with adjustment for risks of overfitting) by adding additional links and removing some of the initially proposed links.

Previous studies (Nilsson, 2014; Nilsson & Strupp-Levitsky, 2016) have shown that humanism and normativism are negatively correlated with each other. Although we did not formulate any explicit hypotheses about their relation, the correlation between these two latent variables was included in the model, and was expected to be negative, in line with the underlying theory and previous results.

The model is built on the following hypotheses:

H1. Personal values and core political values (as indicators) can be positively predicted from four value orientation factors (as latent variables), with higher ratings of personal values and core political values indicating higher importance of the corresponding value orientation factor: (i) Self-transcendence (including the basic personal values of universalism and benevolence, and core political values of equality, civil liberties, accepting immigrants, and concern for the environment), (ii) Conservation (including the basic personal values of tradition, conformity, security, and core political values of blind patriotism, traditional morals, law and order, free enterprise, and military intervention), (iii) Self-enhancement (including the basic personal values of power and achievement), and (iv) Openness to change (including the basic personal values of hedonism, stimulation, and self-direction).

H2. Conservation positively predicts socially/culturally conservative political orientation, with higher conservation corresponding to higher social conservatism, and openness to change negatively predicts socially/culturally conservative political orientation, with higher openness to change predicting lower social conservatism. Self-enhancement and self-transcendence are unrelated or weakly related to socially/culturally conservative political orientation.

H3. Self-enhancement positively predicts economically rightist political orientation, with higher self-enhancement corresponding to higher rightism, and self-transcendence negatively predicts economically rightist orientation, with higher self-transcendence predicting lower rightism. Conservation and openness to change are unrelated or weakly related to economically rightist orientation.

H4. Humanism positively predicts self-transcendence, with higher humanism predicting higher self-transcendence, and is unrelated or weakly related to openness to change, self-enhancement, and conservation.

H5. Normativism positively predicts self-enhancement and conservation, with higher normativism predicting higher self-enhancement and higher conservation, negatively predicts self-transcendence, with higher normativism corresponding to lower self-transcendence, and is unrelated or weakly related to openness to change.

H6. Humanism and normativism differentially predict political orientations indirectly via value orientation factors. That is, the effect of worldviews on political orientation are mediated by value orientation factors. We do not formulate a strong version of this hypothesis with full mediation because we allow for the possibility that there are also direct negative links from humanism to economically left-right orientation and socially liberal-conservative orientation, and direct positive links from normativism to the two political orientations.

To test the model, we combine in a single SEM analysis some of the data previously reported by Nilsson et al. (2020) with previously unpublished data from the same dataset. In their analysis, Nilsson et al. (2020) reported results on how self-reported political orientation, worldviews, and political values predicted party preferences of Swedish voters but did not examine the interrelations among these predictors. In the analysis reported below, we add measures of importance of basic personal values collected from the same sample to test the six hypotheses defined above.

## Materials and Method

### Sample

Data were collected from a random representative sample from Sweden, with the participant age varying between 18–76 years. The data were collected in spring 2015, in between the general elections in September 2014 and September 2018. The participants filled out a postal survey administered by Statistics Sweden (SCB), which is a public administrative agency. After excluding 51 persons with missing values, participants were *N* = 1294 (50.5% women) with mean age = 52.1 years, *SD* = 16.0. Eighty-eight per cent of the respondents were born in Sweden, 58% were employed or self-employed, 5% were students, and 26% retired. The completed educational levels in the sample were the following: only primary school, 16%, only high school, 29%, post-high school education, 17.5%, and bachelor or master’s degree, 36%. The Regional Ethical Review Board in Stockholm, Sweden (Regionala etikprövningsnämnden i Stockholm) approved the study (approval protocol number 2015/5:2). Participants indicated their consent by opting to complete the survey after being informed about the study.

### Measures

#### Basic worldviews

Humanism and normativism were measured using a reduced 18-item version of Nilsson’s (2014) 30-item scale with 15 humanistic and 15 normativistic items, although data for the full 30-item scale were collected. Sample items included, for humanism, ‘All persons are in themselves valuable’ and, for normativism, ‘Human beings should be loved only when they have acted so that they deserve to be loved.’ The participants indicated their agreement with the items on a seven-point Likert type scale ranging from 1 (‘completely disagree’) to 7 (‘completely agree’). As compared to the 30-item scale, we excluded the three humanistic and the three normativistic items of the corresponding political belief facets due to overlap between the items in this facet and the items assessing core political values. We also excluded the epistemological items for humanism and normativism due to generally very low item-total correlations for these items, as shown by the reliability analysis of the data. Cronbach’s Alpha for the nine-item humanism (0.79) and normativism (0.77) scales was acceptable or good.

#### Basic personal values

The participants completed the 21-item version of the Portrait Values Questionnaire (PVQ, Schwartz, 2006). The PVQ includes 21 short verbal portraits of different people, each describing a person’s goals, aspirations or wishes that point implicitly to the importance of a value, and the respondents are asked to indicate how similar they feel to each described target person. For example, ‘It is very important to him to show his abilities. He wants people to admire what he does’ describes a person who considers achievement values to be important. The PVQ measures each of the 10 motivationally distinct types of values with two or three items. For each portrait, respondents indicate how similar the person is to themselves on a scale ranging from 1 = ‘very much like me’ to 6 = ‘not like me at all’. The PVQ is recommended by Schwartz (2006) as the most appropriate measurement of basic personal values, because its items are concrete and cognitively accessible, which makes the PVQ suitable for use with various segments of the population.

#### Core political values

The participants also completed a modified version of the Core Political Values scale (CPV, Schwartz et al., 2010), measuring nine types of political values: traditional morality, law and order, blind patriotism, foreign military intervention, free enterprise, equality, civil liberties, accepting immigrants, and environmentalism. The original scale measures the former eight values; two items measuring the latter value were added in this study based on a previous adaptation by Dimdins et al. (2016) as a complement to the political values defined by Schwartz et al. (2010). On a five-point Likert type scale ranging from 1 (‘completely disagree’) to 5 (‘completely agree’), the participants had to indicate their agreement with items such as ‘It’s right for the government to take restrictive measures on civil liberties to guarantee the security of citizens’ (law and order) and ‘It is unpatriotic to criticize this country’ (blind patriotism).

#### Political orientation

All respondents indicated on separate single-item scales their level of social conservatism and economic rightism (Dimdins et al., 2016). For social conservatism, the item read, ‘To what extent would you characterize yourself as socially liberal (emphasizing the expression of individual rights and freedoms over the observance of tradition and social norms) or socially conservative (emphasizing the observance of social norms and traditions over the expression of individual rights and freedoms)?’ and the response scale was marked with ‘very liberal’ for 1, ‘middle of the road/moderate’ for 4, and ‘very conservative’ for 7. For economic rightism, the item read, ‘To what extent would you characterize yourself as economically leftist (emphasizing a reduction of economic inequality in a society, even if it leads to redistribution of economic resources) or economically rightist (emphasizing individuals’ rights to reap the full results of their economic success, even if it leads to economic inequality)?’ and the response scale was marked with ‘very leftist’ for 1, ‘middle of the road/moderate’ for 4, and ‘very rightist’ for 7.

### Data Analysis

IBM SPSS Amos (27.0) software was used for fitting a number of different SEM models. The fit of each model was assessed by the Chi-square goodness-of-fit test, with lower values indicating better fit, and by the root mean square error of approximation (RMSEA), which reflects the discrepancy between the hypothesized model, with optimally chosen parameter estimates, and the population covariance matrix. We regarded RMSEA < 0.08, as acceptable fit (Hu & Bentler, 1999). To compare the fit of different models we used two parsimony-adjusted comparative fit indices PNFI and PCFI (Mulaik et al., 1989) which assess the fit in relation to a null model assuming no covariations among involved variables. These measures were used for finding a model which was not victim to overfitting, that is, avoiding a model where added new parameters would result in a distorted model that reflected chance variations in the data.

## Results

Below we first describe how we refined the base model in a number SEM analyses, and in this connection, we checked whether adding direct links from worldviews would not improve the model, as predicted in Hypothesis 6. Thereafter, we examined the structure of the resulting fitted model.

### Refinement of the SEM model

We have assumed that the structural model presented in the introduction (the base model) for how political orientations are guided by value orientations and worldviews would be open for refinement by adding new links between the measured variables. We considered if the following types of new links (i.e., regression weights) could be added: links between value orientation factors and political values, as well as personal values, and also links between value orientation factors. We also checked whether adding links between worldviews and political orientations would improve the fit of the model, hypothesizing that it would not be the case. We assumed zero covariations between error variances, since leaving open the possibility for such covariances could result in a distorted model (Hermida, 2015).

The refinement of the base model was in a first step based on the modification indices provided by the AMOS program, which suggested structural changes that could improve the fit of a given model in line with the Chi-square difference test. We started by fitting the base model and then successively added links one at a time that would result in the largest fit improvement. We stopped adding links when PNFI and PCFI did not increase after adding a new parameter. In a second step, we removed links as long as PNFI and PCFI resulted in increased fit values.

**Figure 2:**
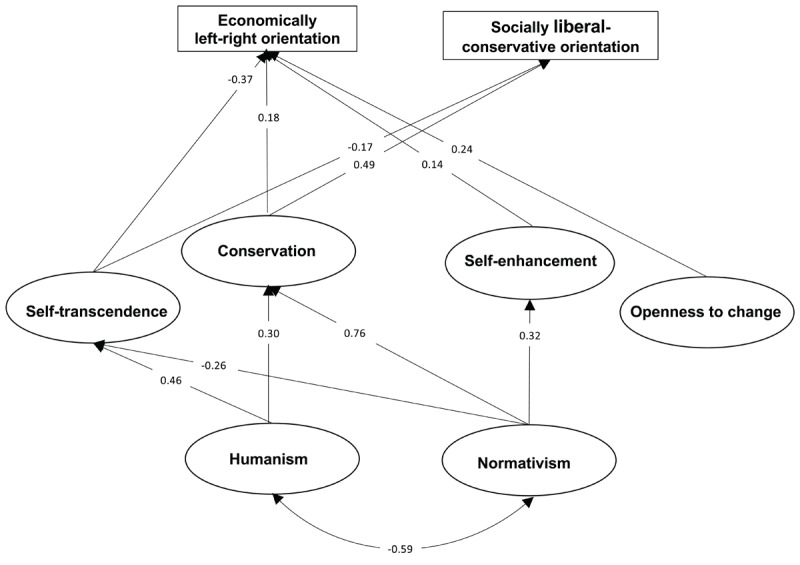
Structural weights in the fitted model as well as weight for links between value orientation factors and political orientations. All coefficients are statistically significant (p < 0.05).

The base line model turned out to yield an RMSEA value of 0.085, which did not meet the acceptability criterion of RMSEA < 0.080 (χ^2^(308) = 3200.85, PNFI = 0.613, PCFI = 0.631). A total of 13 links were added in the refinement process, namely nine links from value orientation factors to personal values, and four links to political values, while one link to political values was removed (conservation to military intervention). The links from the value orientation factors, self-enhancement and openness to change to the socially-liberal-conservative political orientation, were removed. Also removed were the links from humanism to self-enhancement and openness to change, and from normativism to openness to change. Fit measures of the resulting SEM model were χ^2^(302) = 2021.35, RMSEA = 0.066, PNFI = 0.697, PCFI = 0.716. Thus, RMSEA now met the acceptability criterion of RMSEA < 0.080. Adding links from any of the two worldviews to political orientations did not result in a better fit according to the parsimony-adjusted fit measures (PNFI = 0.695, PCFI = 0.716, RMSEA = 0.066). The model resulting from the refinement of the base model is shown in Figure 2, Tables 1 and 2. However, it should be noted that adding a link from normativism to economically left-right orientation resulted in a significant coefficient of –0.16 (*p* < 0.001). On the other hand, the fact that the link is *negative* goes against Hypothesis 6, which expected that there could be a positive link between normativism and economically left-right orientation. There is no good theoretical explanation for this negative link, which together with the lack of model improvement according to the parsimony-adjusted fit measures, led us to conclude that the unexpected link resulted from capitalization of chance variation, and hence, that we could disregard this link in our finally fitted model.

**Table 1:** Standardized regression coefficients in predictions of humanistic and normativistic worldviews from specific indicators. Means and SDs of ratings of items in the indicators in the two rightmost columns.


WORLDVIEW	INDICATOR	REGRESSION COEFFICIENT	*M* ^a^	*SD*

Humanism	Human nature	0.68	5.63	1.11

Humanism	Interpersonal attitude	0.88	5.95	1.02

Humanism	Attitude to affect	0.45	5.30	1.04

Normativism	Human nature	0.66	2.94	1.38

Normativism	Interpersonal attitude	0.84	2.68	1.45

Normativism	Attitude to affect	0.34	4.73	1.21


^a^The means are on 1–7 scale from 1 = Completely disagree to 7 = Completely agree.

**Table 2:** Standardized regression coefficients in predictions of values (rows) from value orientation factors (columns). In italics: core political values. Bold faced numbers: links expected from base model. All coefficients are statistically significant (p < 0.05). Means and SDs of the value ratings in the two rightmost columns.


VALUE	SELF-TRAN-SCENDENCE	CONSER-VATION	SELF-ENHANCE-MENT	OPENNESS TO CHANGE	*M* ^a^	*SD*

**Personal values**						

Universalism	**0.76**			0.28	4.67	0.80

Benevolence	**0.48**			0.39	4.84	0.83

Conformity	0.33	**0.41**	0.45	–0.19	3.76	1.05

Security	0.32	**0.47**			4.21	1.03

Tradition	0.34	**0.54**			3.83	0.99

Power			**0.88**	0.38	2.89	0.94

Achievement			**0.52**	0.21	2.95	1.14

Self-direction				**0.56**	4.40	0.90

Stimulation				**0.64**	3.30	1.15

Hedonism				**0.60**	3.94	1.03

**Political values**						

*Accept immigrants*	**0.45**	–0.41			3.39	1.16

*Equality*	**0.57**				3.63	0.99

*Civil liberties*	**0.34**				4.12	0.81

*Environment*	**0.46**				4.41	0.84

*Traditional morality*		**0.82**			2.37	1.00

*Law and order*		**0.73**			2.35	0.98

*Blind patriotism*		**0.59**			1.91	1.04

*Free enterprise*	–0.22	**0.29**		0.33	2.08	0.99

*Military intervention*				0.19	2.33	1.06


^a^The means for personal values were computed by reversing the scale responses, after reversal going from 1 = Not like me at all to 6 = Very much like me. The ratings of core political values scale went from 1 = Completely disagree to 5 = Completely agree.

### Structure of the Fitted SEM Model

#### Indicators of Latent Variables

The three proposed indicators of humanism and normativism were significantly (*p* < 0.05) and positively linked to the relevant worldview, see Table 1. The strongest links were found for the humanistic and normativistic variants of interpersonal attitude, with coefficients quite close to 1. The humanistic views of human nature and interpersonal attitude were on average strongly endorsed by the participants, whereas the normativistic views tended to be rejected, although the relatively high standard deviation of the normativistic view was concordant with the fact that a minority of the participants endorsed the normativistic views.

Table 2 shows how personal and political values were linked to the four value orientation factors (only significant [*p* < 0.05] links are shown) and also showed means and standard deviations of the ratings of each value. Personal values were all linked to value orientation factors as expected from Schwartz’s value model (see the boldfaced numbers in Table 2). The coefficients were moderately or quite high, ranging from 0.41 (conservation to conformity) to 0.88 (self-enhancement to power). There were a few additional links between personal values and value orientation factors, which all, except for conformity to self enhancement, were weaker than the expected links.

The political values had the expected positive links to self-transcendence and conservation on approximately the same strength level as was true for the three personal values. Note that accepting immigrants was not only positively linked to self-transcendence, as expected, but also negatively linked to conservation. In contrast to the structure in the base model, where only self-transcendence and conservation were linked to political values, also openness to change had links to free enterprise and military intervention. In line with expectations, self-enhancement was not significantly related to any of the political values.

#### Prediction of Value Orientation Factors from Worldviews

As can be seen in Figure 2, humanism and normativism were negatively correlated (*r* = –0.59), which implied that we could expect different patterns of links from the worldviews to the value orientations. Indeed, we could see that humanism mainly predicted self-transcendence (regression coefficient = 0.46), whereas normativism strongly predicted conservation (regression coefficient = 0.76) and less strongly negatively predicted self-transcendence (regression coefficient = –0.26). Conservation was also, but less strongly positively (regression coefficient = 0.30) predicted by humanism. This link, which was not expected, could be understood from the fact that some indicators of self-transcendence and conservation covaried positively with each other (e. g., benevolence correlated 0.23, 0.14, and 0.20 with tradition, conformity and security, respectively).

Self-enhancement was predicted, although quite weakly (regression coefficient = 0.32), by normativism. In line with expectations, openness to change was not predicted from any of the two worldviews. Thus, self-transcendence and conservation were much more strongly related to the two worldviews than was true for self-enhancement and openness to change. This is illustrated in the multiple correlations in predictions in the fitted SEM model of each of the four value orientation factors from humanism and normativism, being 0.64 for self-transcendence, 0.63 for conservation, and 0.32 for self-enhancement and 0 for openness to change (as a result of there being no link to this value orientation factor in the fitted model).

#### Prediction of Political Orientations

We examined how well the fitted model predicted the economically left-right orientation and the social liberal-conservative orientation. To understand this, it was important to look at how participants rated these two orientations. The correlation between the two political orientation measures was quite low (*r* = 0.27) and, hence, to a large extent probably tapped different aspects of the participants’ political orientation. The ratings of political orientations also differed with respect to their means and standard deviations, indicating a mean that was closer to the liberal than to the conservative pole for the socially liberal-conservative orientation (*M* = 3.35) whereas the mean was just in between the two poles of economically left-right orientation *M* = 4.06), and with a lower standard deviation for ratings of socially liberal-conservative orientation (*SD* = 1.42) than for economically left-right orientation (*SD* = 1.65).

We then considered how the value orientation factors predicted political orientations. Both political orientations had in common that they were predicted via negative links from self-transcendence and positive links from conservation. However, the strength of these links differed between political orientations, with the link from self-transcendence being stronger for economically left-right orientation, and the link from conservation being much stronger for socially liberal-conservative orientation. This imbalance between the two links was more pronounced for socially liberal-conservative orientation, since the leading link here (from conservation) was stronger than it was for the leading link to economically left-right orientation (from self-transcendence). Another difference between the two orientations was that economically left-right orientation also was (positively) linked to self-enhancement and openness to change. Overall, both political orientations were quite well predicted from the value orientation factors, multiple correlations in the fitted SEM model being 0.55 for economically left-right orientation, as well as for socially liberal-conservative orientation.

How do worldviews predict political orientations? In the fitted model, the effects of worldview on political orientations were indirect effects mediated by value orientation factors. In line with this, we have already seen that when taking parsimony-adjusted fit measures into account, there was no support for non-mediated effects from worldviews on political orientations. The indirect effects presented in Table 3 showed that normativism was a better predictor than humanism of both economically left-right orientation and socially liberal-conservative orientation.

**Table 3:** Indirect effects of worldviews on ratings of political orientations.


WORLDVIEW	ECONOMICALLY LEFT-RIGHT	SOCIALLY LIBERAL-CONSERVATIVE

Humanism	–0.11	0.07

Normativism	0.28	0.42


## Discussion

On a general level, the results support our base model for how worldviews, value orientation factors and political orientations are related to each other in the investigated sample of Swedish residents. Structural equation modelling showed that socially liberal-conservative orientation and economically left-right orientation could be predicted from humanism and normativism, mediated by value evaluation factors (Hypothesis 6). Furthermore, the base model is on the whole supported with respect to how the observed variables are linked to latent variables in the model (Hypothesis 1). The SEM analysis confirmed all the hypothesized links from value orientation factors to basic values and core political values except one (the core political value of military intervention was not significantly related to conservation, as predicted). It can be said that overall Hypothesis 1 was confirmed, although a number of previously not hypothesized links were found in the final model. In addition to the hypothesized links, it was found that self-transcendence values of universalism and benevolence were also significantly related to the openness to change value factor, which is adjacent to self-transcendence in the value circumplex, and, just like self-transcendence, represents a growth focus. The conservation values of security and tradition were related to self-transcendence. Both self-transcendence and conservation have a social focus and are adjacent in the value circumplex. Self-enhancement values of power and achievement were also related to the openness to change factor, which also has a personal focus. Finally, the conformity value was significantly related to all four value orientation factors – conservation (in line with Hypothesis 1), self enhancement (reflecting its stability-focus), self-transcendence (reflecting its social focus), and, negatively, to openness to change (a personal- and growth-focus value factor).

Both the hypothesized and additional findings are fully in line with Schwartz’s theoretical model of basic personal values (Schwartz et al., 2012). For core political values, very few unpredicted links emerged – accepting immigrants was negatively related to conservation, military intervention was positively related to openness to change, and free enterprise was also positively related to openness to change and negatively related to self-transcendence. These additional links probably reflect the specific context of Swedish political culture, but overall the relation between core political values and value orientation factors in our model is in line with the results reported by Schwartz et al. (2014) and confirms our theoretical model. These results extend and complement previous findings about the relationship between basic personal values and core political values (Caprara et al., 2009; Schwartz et al., 2010; Schwartz et al., 2014), indicating that both constructs share a common value orientation base, and can be seen as expressions of the same psychological inclinations in more general (in case of basic personal values) versus more specific (in case of CPVs) contexts. In this way our findings contribute to research examining the psychological underpinnings of political orientation (in particular, on how basic values and CPVs predict political orientation), and to the literature on human values by illustrating the broad motivational role that values play in human judgments across various domains.

Hypothesis 2, which concerned links from value orientation factors to liberal versus conservative political orientation, was supported regarding two of its predictions – we found evidence for a link from conservation values to liberal-conservative political orientation, and no significant link from self-enhancement to liberal-conservative political orientation. Yet Hypothesis 2 was rejected in terms of the two other predictions – we did not find a significant negative link from openness to change to liberal-conservative orientation, and we found a significant positive link from self-transcendence to liberal-conservative orientation. Hypothesis 3, which concerned links from value orientation factors to economically left-right orientation, was also only partially supported, with the hypothesized significant positive link from self-enhancement and the hypothesized negative link from self-transcendence to economically left-right orientation present in the fitted model, but significant, not hypothesized, positive links from openness to change and conservation to economically left-right also emerged in the results. These results substantially contribute to the empirical knowledge base on the relationship between basic values and political orientation (Caprara et al., 2009; Schwartz, 1994; Schwartz et al., 2010; Schwartz et al., 2014) by showing that the general value orientation factors forming the end-poles of the two value dimensions are systematically related to the socio-cultural and economic dimensions of political orientation, but do not overlap with these dimensions as suggested in previous literature (c.f. Dimdins et al., 2016; Schwartz, 1994).

To get an overall view of the structure of the fitted SEM model, it is fruitful to examine the mediating role of each of the four value orientation factors. We found that self-transcendence and conservation were the main mediating value orientation factors. They were quite strongly related to worldviews and had relatively strong links to political orientations, demonstrating that both dimensions are separate, but not orthogonal. It may be concluded that both dimensions of political orientation can be seen as to a large extent based on the same underlying value factors, but with more emphasis on self-transcendence for the economic dimension, and more emphasis on conservation for the social dimension. This is in line with the previous theoretical reasoning (Schwartz, 1994) and empirical findings (Dimdins et al., 2016) on the relationship between the two dimensions of political orientation and both value dimensions.

Two value orientation factors that are adjacent to each other in Schwartz’ value circle – self-transcendence and conservation – played a key role, both as predictors of political orientations, and in being themselves predicted from worldviews. This pattern can be seen as being consistent with Schwartz’s value theory (Schwartz, 1992; 1994; Schwartz et al., 2012). Both self-transcendence and conservation have in common their social focus, in contrast to self-enhancement and openness to change, which both have a personal focus. Presumably, a social focus is more relevant for political orientation than is true for a personal focus, which may primarily reflect a more apolitical, personal sphere of people’s lives. Schwartz (2006) and Schwartz et al. (2012) further assert that conservation and self-transcendence contrast with each other with respect to self-protection against threat (conservation) and self-expansion and growth (self-transcendence), as is also true for the two personally focused value poles.

Hypothesis 4, which concerned links from humanism to value orientation factors, was partially supported, with a positive link found between humanism and self-transcendence, and no significant links between humanism and self-enhancement and openness to change, but we found an unpredicted significant positive link from humanism to conservation. Hypothesis 5 on links between normativism and value orientation factors was fully supported, with normativism positively predicting self-enhancement, and conservation negatively predicting self-transcendence, and being unrelated to openness to change. Our findings strongly suggest that worldviews constitute a major underlying factor in explaining the polarization between liberal-conservative and left-right political orientations. These findings are in line with, and complement, the results reported by Nilsson and Jost (2020), who found that humanism predicted political orientation through a strong positive relationship with preference for equality, and normativism predicted political orientation through a positive relationship with resistance to change. We show that humanism is related to economically left-right orientation through the importance of self-transcendence values (with their growth focus), and normativism is related to social conservatism through the importance of conservation values (with their protection focus). It should be noted that humanism is positively related to both self-transcendence and conservation factors. The possible explanation for this pattern is that both self-transcendence and conservation are socially oriented value orientation factors, and humanism, with its focus on human growth and mutual care might share a significant variance with both value factors related to this common concern for human well-being. Our results offer additional insight and empirical support for the theorized role of basic worldviews as central axes organizing the social and political belief systems of individuals (Jost et al., 2003; Tomkins, 1995).

It should be noted that openness to change, which according to Schwartz (2006) and Schwartz et al. (2012) has a personal focus, in our results plays a role in predicting an economically left-right orientation. The reason for this link, which is also in opposition to Hypothesis 3 (that self-enhancement rather than openness to change predicts economically left-right orientation) may be that the core political value free enterprise was linked to openness to change, presumably because a minimally regulated economy (economic rightism) allows for a greater (positive or negative) change in economic enterprise.

Our results show that normativism in general is a more powerful predictor of value orientation factors than is true for humanism. This could be related to the fact that humanism on average was highly appreciated whereas the normativistic items tended to be less positively evaluated and to vary more across participants than was true for humanism. This pattern may reflect that humanism more than normativism is in line with dominant norms in Sweden as a result of the long history of Social Democratic rule in this country with its emphasis on welfare and social and economic equality (Nilsson & Jost, 2020). The same may be true for self-transcendence, which shows less variation compared to the other three value orientation factors. In other words, the respondents generally showed higher and less variable scores on humanism and self-transcendence value ratings (a pattern possibly representing a social desirability effect reflecting the prevalent social norms in the Swedish society), resulting in lower covariation between the two latent variables, but they varied more in normativism and conservation value scores, leading to higher covariation between the two.

Our findings contribute to several lines of literature. First, they provide additional insights about the structure of political orientation. Our results do not support the idea that the two primary value dimensions in Schwartz’s circumplex – openness to change versus conservation and self-transcendence versus self-enhancement – correspond to orthogonal dimensions of social and economic political orientation, respectively (c.f. Schwartz, 1994). Instead, we found that both dimensions of ideology are strongly predicted by the same two poles, representing both value dimensions (self-transcendence and conservation). Thus, our results demonstrate that both dimensions are separate, but not orthogonal, in line with numerous findings where both dimensions have been found to be highly correlated (Malka et al., 2014). Our findings also illustrate the role of secondary value dimensions in explaining political orientation – political orientation was much more strongly predicted by social-focus values, and only weakly predicted by personal-focus values. The results also illustrate the mediating role of values between basic worldviews and political orientation. Previously, basic personal values have been found to mediate the relation between personality traits and political orientation (Caprara et al., 2009); our findings suggest that values may have a wider, more universal function in transforming basic psychological traits and individual differences into political preferences. Our second contribution is the knowledge of how basic personal values are related to core political values, where our results confirm previous findings, showing that core political values are primarily associated with self-transcendence and conservation values (Schwartz et al., 2010; Schwartz et al., 2014). Third, our findings extend and complement previous research on the relation of basic worldviews to political orientation and to basic personal values (Nilsson & Strupp-Levitsky, 2016; Nilsson & Jost, 2020), showing an asymmetric pattern where normativism predicts conservation values and self-transcendence values with opposite signs, whereas humanism is positively related to both value orientation factors.

The results are based on a representative sample and thus can be generalized to the Swedish population. Given the similarities with previous findings, these results might be cautiously generalized to other liberal democracies representing political cultures with high ideological constraints, where the public discourse systematically pronounces how political attitudes and beliefs should be organized within certain ideologies, although more research is needed to test the generality of the model. It is less clear whether the same pattern of relationships would be found in political cultures with low ideological constraints, where the relationships among political concepts and ideas (and how these concepts and ideas link to basic values and worldviews) are less clearly prescribed in the political discourse (Malka et al., 2014). In addition to cross-cultural testing of the model, future research should focus on several promising directions. One would be replication of findings with different measures of the same constructs, such as by using alternative measures of value importance, or political orientation to test the reliability of the findings and their generalizability to psychological theory. Another promising direction would be to develop the model by adding other relevant constructs that have been shown to correlate with values, worldviews, and political orientations, such as social dominance orientation, right-wing authoritarianism, system justification, the importance of moral foundations, and others (Dimdins et al., 2016; Nilsson & Jost, 2020). In addition, the role of other constructs representing fundamental psychological tendencies that have been suggested to constitute the broader motivational basis of political orientation – such as uncertainty avoidance, need for cognitive closure, or regulatory focus as a general orientation (see Jost et al., 2003) – should be investigated alongside, and in conjunction with, basic worldviews as predictors of value orientation factors and political orientations. Third, the mediating role of values suggests possibilities for experimental studies that would test the role of value activation in political judgment and political behavior (c.f. Verplanken & Holland, 2002).

In conclusion, the present study has illustrated how a framework involving worldviews (humanism or normativism) and clusters of core political and basic personal values may be used for studying the psychological underpinnings of political orientation. Mapping the psychological mechanisms behind political orientation leads to better understanding of political polarization and conflict and may help identifying and presenting solutions that respect and integrate the fundamental values and motives of all parties involved. It may also help to better frame and convey messages to partisan groups by addressing values and worldviews important to these groups, so that people with diverse ideological views are engaged in solving of socioeconomic problems, such as poverty or environmental issues. Our findings illustrate the importance of approaching the study of political orientation from various theoretical and methodological perspectives to find the best way of using and combining the existing theoretical models to explain the psychology of political ideology.

## Data Accessibility Statement

The data file is freely and publicly available from the Open Science Framework by following this link https://osf.io/mhc3z/.

## Author Note

Part of the data presented here (measures of self-reported political orientation, humanism, normativism, and core political values) has been published in a paper focused on the psychological profiles of Swedish voters in the European Journal of Personality (https://doi.org/10.1002/per.2249).
